# Mindfulness augmentation for anxiety through concurrent use of transcranial direct current stimulation: a randomized double-blind study

**DOI:** 10.1038/s41598-021-02177-3

**Published:** 2021-11-23

**Authors:** Keiichiro Nishida, Yosuke Morishima, Roberto D. Pascual-Marqui, Shota Minami, Tomonari Yamane, Masahito Michikura, Hideki Ishikawa, Toshihiko Kinoshita

**Affiliations:** 1 Department of Neuropsychiatry, Kansai Medical University, 10-15 Fumizono-cho, Moriguchi, Osaka 570-8506 Japan; 2 Translational Research Center, University Hospital of Psychiatry, University of Bern, Bolligenstrasse 111, 3000 Bern, Switzerland; 3 The KEY Institute for Brain-Mind Research, University Hospital of Psychiatry, Postfach 1931, 8032 Zurich, Switzerland; 4 Graduate School of Psychology, Kansai University, 3-3-5 Yamate-cho, Suita, Osaka 564-8680 Japan; 5 Medical Research Support, 3-1-14 Koraibashi, Osaka, Osaka 541-0043 Japan; 6 Department of Molecular-Targeting Cancer Prevention, Graduate School of Medical Science, Kyoto Prefectural University of Medicine, 3-2-17-2F Imabashi, Chuo-ku, Osaka, Osaka 541-0042 Japan

**Keywords:** Prefrontal cortex, Emotion, Neuroscience, Stress and resilience, Health care, Disease prevention

## Abstract

Transcranial direct current stimulation (tDCS) have revealed the capability to augment various types of behavioural interventions. We aimed to augment the effects of mindfulness, suggested for reducing anxiety, with concurrent use of tDCS. We conducted a double-blind randomized study with 58 healthy individuals. We introduced treadmill walking for focused meditation and active or sham tDCS on the left dorsolateral prefrontal cortex for 20 min. We evaluated outcomes using State-Trait Anxiety Inventory-State Anxiety (STAI) before the intervention as well as immediately, 60 min, and 1 week after the intervention, and current density from electroencephalograms (EEG) before and after the intervention. The linear mixed-effect models demonstrated that STAI-state anxiety showed a significant interaction effect between 1 week after the intervention and tDCS groups. As for alpha-band EEG activity, the current density in the rostral anterior cingulate cortex (rACC) was significantly reduced in the active compared with the sham stimulation group, and a significant correlation was seen between changes in STAI-trait anxiety and the current density of the rACC in the active stimulation group. Our study provided that despite this being a one-shot and short intervention, the reduction in anxiety lasts for one week, and EEG could potentially help predict its anxiolytic effect.

## Introduction

Anxiety is a defensive ability to avoid danger that is essential to the process of natural selection, helping the chance of survival. However, when anxiety and fear are excessive, lacking, or inadequate, adaptation is difficult^1,2^. Continuous exposure to threats, fear, and anxiety are more likely to cause mental health problems such as anxiety disorders and depression^3^. Therefore, it has been suggested that individuals need to develop their ability to cope with mental stress in advance^4^.

In this context, mindfulness meditation is gaining attention for its ability to calm the hyperactivity that is increasingly common in modern society. Mindfulness-based stress reduction and mindfulness-based cognitive therapy (MBCT) have become widely accepted medical interventions for anxiety disorders and depression in the recent decade^5,6^. However, these skills can take weeks and sometimes years to acquire. To address this problem, a recent study aimed to facilitate the early acquisition of mindfulness through the use of transcranial direct current stimulation (tDCS)^7^.

tDCS is a non-invasive brain stimulation method that uses a simple and inexpensive device to modify neuronal excitability by delivering weak direct current, about 1–2 mA, through electrodes placed on the scalp^8^. In the last decade, tDCS has been the subject of an increasing number of papers published for both basic and clinical research^8–12^.

In general, meditation can be divided into focused and insight meditation^13^. Focused meditation regulates the occurrence of mind-wandering. Mind-wandering is a state of mind where one drifts into thoughts that have no relation to the present. In recent years, cognitive science has revealed a link between mind-wandering and psychiatric illness, including anxiety disorders, depression, and attention deficit hyperactivity disorder^14^. Anxiety is often followed by anxious content of the mind-wandering^15^, and thus, it is important to prevent the occurrence of mind-wandering.

Focused meditation essentially requires a restful environment free of distractions. However, the standard tDCS approach can easily interfere with concentration meditation because of the sensitivity of the skin on the head. We looked specifically at walking mindfulness, which focuses the senses on the feet, away from the head^16^. To maximize the benefits of walking mindfulness, we developed a method known as treadmill walking for focused mindfulness (TW-FM). Treadmills allow individuals to walk slowly at a constant speed in the same spot in a room. This allows people to walk safely and avoid collisions, and reduces the burdensome tDCS equipment that disrupts meditation.

Electroencephalograms (EEGs) were introduced as an objective measure of the effects of TW-FM. Focused meditation is thought to be related to the brain’s attentional function because it focuses attention on the present moment of an object. From a neurophysiological perspective, the alpha-band component of an EEG is thought to be involved in the allocation of attentional resources^17–19^. Alpha rhythms have long been studied in Zen meditation^20^. In the last few years, some studies have also used mindfulness to investigate alpha activity^21^. Here, we focused our attention on the rostral anterior cingulate cortex (rACC), which is thought to play an important role in depressive disorders. Recent EEG-based studies suggest that the rACC is the main area that predicts response to antidepressants^22^. EEGs can measure brain function before and after an intervention without changing the study environment, so they are more sensitive to the time resolution of changes compared with functional magnetic resonance imaging or positron emission tomography.

Therefore, the purpose of this randomized, double-blind, sham-controlled study was to investigate the augmentation effect of mindfulness. To this end, we introduced TW-FM to healthy adult individuals and applied active or sham tDCS on the left dorsolateral prefrontal cortex for 20 min during TW-FM. We evaluated outcomes using the State-Trait Anxiety Inventory (STAI)-State Anxiety^23^ before the intervention and immediately, 60 min, and 1 week after the intervention, as well as current density from EEGs before and after the intervention.

## Materials and methods

### Study design

We performed a randomized double-blind study to compare changes in active and sham tDCS groups under TW-FM. This study was conducted at the Kansai Medical University Medical Center, Osaka, Japan. The study was approved by the Certified Review Board of Hyogo College of Medicine (CRB51800005; date of approval: 15 January 2019). The data were collected from 1 February 2019 to 31 May 2020. No changes were made to the study protocol after approval.

### Participants

Sixty right-handed healthy individuals (age range, 20–60 years) participated in this study. The exclusion criteria were current or previous history of psychiatric or neurological disorders, current or previous metal (except titanium) implants (e.g., in the brain or skin), current or previous metal equipment in the body (e.g., a pacemaker), a history of head surgery, current or previous head injuries and/or resultant disorders of consciousness, skin problems, a history of epileptic seizures, a history of syncope, pregnancy or possible pregnancy, current drug user, and tDCS and TMS experience. Psychiatrists with more than 3 years of experience in the field confirmed the absence of previous neurological and psychiatric disorders through semi-structured interviews.

The study participants were recruited through posters on the staff bulletin board of Kansai Medical University and related institutes. Prospective participants were initially pre-screened by telephone or email, and then interviewed by an experienced psychiatrist. Written informed consent was obtained from all participants. All procedures in this study were performed in accordance with the Declaration of Helsinki and the guidelines for low-intensity tDCS^24^.

### Randomization and blinding procedures

The statistical analyst of our team assigned participants using a method that minimizes stratification factors. The included stratification factors were: age on the day of consent (20–39 and 40–60 years), gender, and STAI-State Anxiety (STAI-SA) score (< 40, and ≥ 40).

After obtaining consent for participation and confirming eligibility, each individual was registered as a study participant. A registration number was assigned to the participant by the data management centre. For blinding to the active or sham stimulation, we used the “STUDY mode” in DC Stimulator Plus (NeuroConn GmbH, Ilmenau, Germany). In this mode, 5-digit number codes are linked to either the active or sham stimulation, and the experimenter cannot identify which stimulation type is applied. The physician who conducted the experiments was informed of the 5-digit code by the data management centre. The details of the tDCS protocols, such as current intensity, duration, and electrode montages, are described below.

Two physicians, including a medical doctor, administered the tDCS intervention. Upon the completion of data collection, the change rate in rACC activity was calculated before disclosure of the allocation table and sent to the data management centre to fix the data concerning the primary result. The data management centre was responsible for locking the data table. Further analyses were performed after the disclosure of the allocation table.

### Timeline of the intervention and measurements

**Figure 1:**
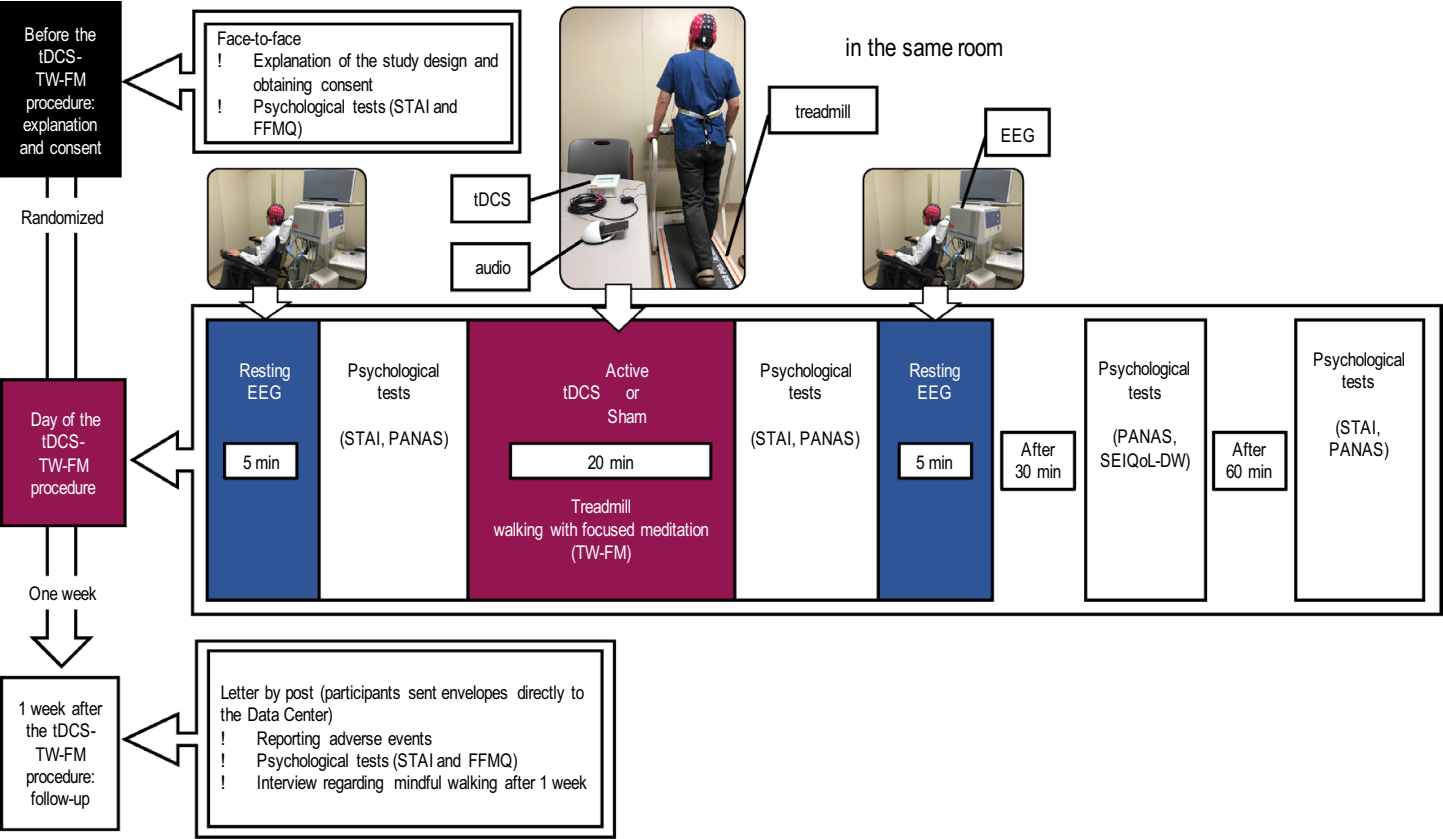
Timeline of the intervention and measurements. *STAI* State-Trait Anxiety Inventory, *EEG* electroencephalogram, *tDCS* transcranial direct current stimulation, *TW-FM* treadmill walking for focused mindfulness, *FFMQ* Five Facet Mindfulness Questionnaire, *PANAS* positive and negative affect schedule.

On the day of the second appointment, we first measured resting-state EEG with the eyes closed and then conducted psychological tests. Next, we carried out TW-FM with either active or sham-tDCS. After tDCS and TW-FM, we remeasured resting-state EEG with the eyes closed and conducted further psychological tests (Fig. 1). All interventions and EEGs were performed and collected at the same laboratory in Kansai Medical University Medical Center.

To assess anxiety, affect, quality of life, and mindfulness status, we conducted a series of psychological tests: STAI, the Positive and Negative Affect Schedule (PANAS)^25^, the Schedule for the Evaluation of Individual QoL-Direct Weighting (SEIQoL-DW)^26^, and the Five Facet Mindfulness Questionnaire (FFMQ)^27^. We conducted these tests at the following time points: at consent (STAI and FFMQ), before tDCS and TW-FM (STAI and PANAS), immediately after tDCS and TW-FM (STAI and PANAS), 30 min after tDCS and TW-FM (PANAS and SEIQoL-DW), and 60 min after tDCS and TW-FM (STAI and PANAS). After the tDCS and TW-FM intervention, the participants were encouraged to engage in walking mindfulness when possible for 1 week, and then to take psychological tests (STAI and FFMQ) and complete a questionnaire on the frequency of walking mindfulness during the week after the intervention. We asked the participants to return the STAI, FFMQ, number of days engaged in walking mindfulness, adverse effects after the intervention, and the onset of any medical conditions to the data management centre by postal mail.

### TW-FM

We used a treadmill (Flat Walker 3914neo; Alinco Incorporated, Osaka, Japan) for the TW-FM. First, participants were instructed about the procedure of TW-FM. Participants were orally instructed about the procedure while being shown a short written brochure. They were told to focus on their soles and thighs in terms of their minutiae and to walk as follows: “First, raise your right heel, but keep your toes on the ground and feel your weight shifting towards your left thigh. Second, raise your right toe and fully shift your weight onto the left thigh. Then, touch your right heel to the ground and prepare to raise your left heel, then shift your weight again towards the right thigh. Lastly, raise your left heel, but keep your toes on the ground and feel your weight shifting towards your right thigh.”

After the instruction and brief practice on the treadmill, the participants walked slowly on the treadmill (speed, 1 km/h). While walking, the participants heard the audio instruction: “While walking, focus on the sense of your sole. Heel to toe, heel to toe. Focus on shifting your weight from one thigh to another. Then, any thought occurring will disappear as you walk”. This instruction was repeated every 2 min to allow them to focus on their soles and have their thoughts spontaneously disappear. At the end of the TW-FM session, the participants were instructed about at-home walking mindfulness. They were encouraged to work on walking mindfulness either at home or outside when they were comfortable doing it. They also took home the written brochure describing the procedure explained during the TW-FM session.

### tDCS

We administered tDCS using a battery-driven stimulator (DC Stimulator Plus; NeuroConn). A circular anodal electrode with a diameter of 5 cm coated with EEG gel was attached to the left dorsolateral prefrontal cortex (DLPFC; F5, International 10/10 system), and a rectangular cathodal electrode (5 cm × 7 cm) was attached to the left shoulder. We chose the current electrode montage instead of placing the anode electrode on the F3 position of 10–20 systems, based on the previous findings that the more anterior portion of the DLPFC could be more effective for repetitive transcranial magnetic stimulation (rTMS) treatment of major depressive disorder (MDD)^28^, and F5 can be closer to the DLPFC target^29^. For the active stimulation condition, we applied 1 mA of tDCS for 20 min, with 30 s of the initial ramp-up and last ramp-down periods to reduce skin sensations due to rapid changes in the current intensity. For the sham stimulation condition, only 30 s of 1 mA tDCS was applied before and after 30 s of the ramp-up and ramp-down periods.

### EEG data recording and analysis

Eyes-closed resting-state EEGs were recorded using an EEG-1200 Nihon Kohden (Neurofax 1200, Tokyo, Japan) system. We used 30 Ag/AgCl sintered ring electrodes (Waveguard; ANT-Neuro, Enschede, Netherlands). Data were sampled at 500 Hz, and recorded with a low-pass filter at 0·5 Hz and a high-pass filter at 60 Hz. The recordings lasted 5 min and were carried out twice for each participant.

For processing, raw EEG data were bandpass filtered at 1–30 Hz. EEG fragments contaminated with muscle artifacts and eye blink noise were excluded by visual inspection. After removing the contaminated fragments, we used an artifact detector in LORETA-KEY software (http://www.uzh.ch/keyinst/loreta.htm) ^30^ and distinguished possible artifacts autonomously. We adopted 2-min epochs with as few artifacts as possible in each session.

Current densities were calculated from the EEG data using standardized low-resolution brain electromagnetic tomography (sLORETA)^30^. The region of interest (ROI) for the rACC (x = 0, y = 45, z = 0 in the Montreal Neurological Institute space) was selected based on a previous study investigating neurophysiological mechanisms in patients with MDD^22^. The ROI for the DLPFC is defined by the location under the stimulation site, based on the 10–10 EEG coordinates (x = –52, y = 26, z = 28)^31^. We analysed the alpha band between 8.5 and 13 Hz.

### Statistical analysis

Based on preliminary results from previous studies, we estimated the relative risk of EEG responders between the active and sham tDCS groups and set the target number of participants at 60 in total (30 for each group)^32^.

STAI-SA, STAI-trait anxiety (STAI-TA), PANAS-Positive Affect (PANAS-PA), and PANAS-Negative Affect (PANAS-NA) scores were analysed using linear mixed-effects models (LMMs) in R version 4·0·2 (R Core Team, 2020) and lme4 package version 1·1-23. As estimates of EEG source analysis yield non-parametric distribution across subjects, we used non-parametric approaches.

Firstly, STAI-SA, STAI-TA, PANAS-PA, and PANAS-NA scores at four fixed time points were analysed using a mixed model. The dependent variable was each of the scores. We included group assignment (Group), time of assessment (Time), and interaction between group and time point (Group * Time) as a fixed effect. The assessment time point was the baseline score just prior to the intervention; for the fixed-effect variables, all dummy variables were created. Group variables were given 1 for the active and 0 for the sham stimulus group so that the coefficients represented the difference from the active stimulus group. The time point of the assessment was treated as a categorical variable, with the coefficient representing the difference from the assessment immediately before the intervention. For SEIQoL-DW and FFMQ scores, we performed the Mann–Whitney test. Significance was defined as p < 0·05.

Next, the rates of change of current densities throughout the intervention were compared between the active and sham stimulation groups. Additionally, the current densities in pre- and post-stimulation were compared in the active and sham stimulation groups, respectively.

As an exploratory analysis, we examined the association between anxiety and neurophysiological response to tDCS-TW-FM. We correlated the ratios of change in STAI scores and rACC current densities from before and after tDCS-TW-FM in the active and sham groups, respectively. To examine this correlation, we used Spearman’s rank correlation because of non-parametric distribution of the EEG source.

### Adverse events

To monitor adverse events, following safety guidelines, we assessed headaches and skin symptoms such as itching, pain, burning, warmth, and others (e.g., metallic taste, fatigue) immediately and at 1 week after the tDCS-TW-FM intervention^24^.

### Data monitoring committee

Data monitoring and management was conducted by Medical Research Support Ltd. The study protocol was reviewed and approved by the Clinical Research Review Committee of Hyogo Medical University (CRB5180005), and the study was registered with the Japan Registry of Clinical Trials (jRCTs052180043, 30/01/2019).

## Results

The registration period was from 2 February to 17 May 2019, and the tDCS-TW-FM intervention period was 10 February to 5 June 2019.

A total of 60 participants were enrolled and underwent randomization; two participants who were allocated to the active group withdrew before undergoing the intervention (active stimulation, n = 28; sham stimulation, n = 30) (Supplementary Fig. S1 online). The participants’ baseline characteristics are shown in Data A (Table 1). The characteristics of the participants, excluding withdrawals and those who failed to submit information at 1 week or had a low-quality EEG, are shown in Data B (Table 2). We adopted Data B for the analyses.

**Table 1:** Baseline characteristics of the participants after excluding those who had low EEG quality data (Data A).

	Overall (*N* = 56)	Active tDCS + TW-FM group (*N* = 27)	Sham tDCS + MW group (*N* = 29)
Age, years, mean (SD)	39.80(10.06)	38.29 (11.34)	40.59 (9.27)
**Sex**			
Male (%)	22 (39)	12 (44)	10 (34)
Female (%)	34 (61)	15 (56)	19 (66)
Education Period (SD)*	14.82 (1.53)	15.09 (1.54)	14.61 (1.52)
**STAI-SA score, mean (SD)**			
Time of consent	39.45 (7.01)	38.04 (6.44)	40.76 (7.38)
**STAI-TA score, mean (SD)**			
Time of consent	44.70 (9.54)	42.82 (7.86)	46.45 (10.72)

*Total 51 cases, missing (4 cases in the Active group and 1 case in the Sham group).

**Table 2:** Baseline characteristics of the participants except those who failed to submit information at 1 week (Data B).

	Overall (*N* = 54)	Active tDCS + TW-FM group (*N* = 26)	Sham tDCS + TW-FM group (*N* = 28)
Age, years, mean (SD)	39.91 (9.92)	39.54 (10.75)	40.25 (9.26)
**Sex**			
Male (%)	21 (39)	12 (46)	9 (32)
Female (%)	33 (61)	14 (54)	19 (68)
Education period (SD)	14.86 (1.50)	15.05 (1.56)	14.70 (1.46)
**STAI-SA score, mean (SD)**			
Time of consent	39.54 (7.03)	37.85 (6.49)	41.11 (7.26)
**STAI-TA score, mean (SD)**			
Time of consent	44.48 (9.65)	42.46 (7.79)	46.36 (10.91)

*STAI-SA*  State-Trait Anxiety Inventory-State Anxiety, *STAI-TA* STAI-Trait Anxiety, *FFMQ* Five Facet Mindfulness Questionnaire, *tDCS* transcranial direct current stimulation, *TW-FM* treadmill walking for focused mindfulness.

*Total 49 cases, missing (5 cases in the Active group and 2 cases in the Sham group).

Supplementary Table S1 online shows a summary of the psychological tests and the number of days the participants engaged in slow walking with mindfulness.

**Figure 2:**
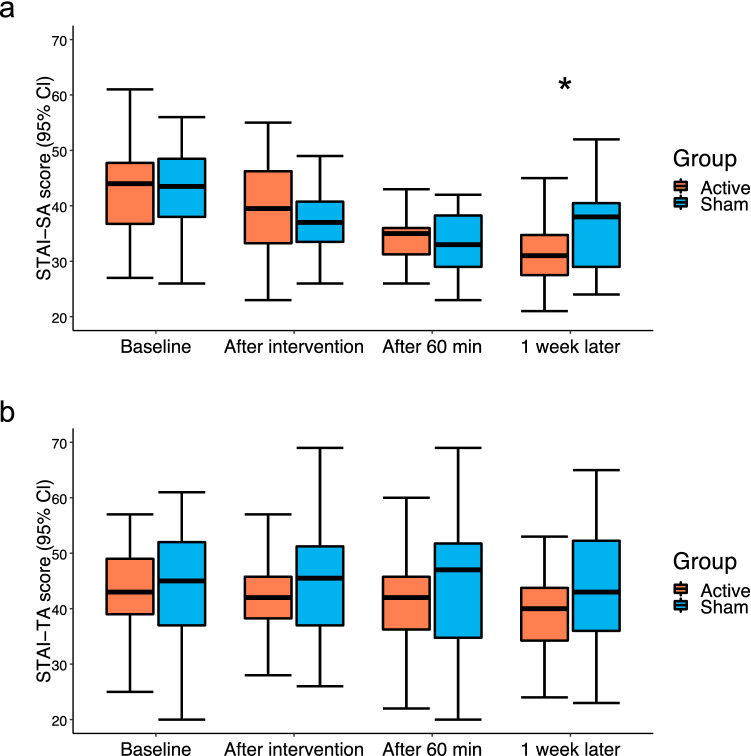
Changes in (**a**) State Trait Anxiety Inventory-State Anxiety (STAI-SA) scores and (**b**) State Trait Anxiety Inventory-Trait Anxiety (STAI-TA) scores. (**a**) A significant interaction effect was observed between 1 week after tDCS (Time) and active or sham tDCS (Group). (**b**) No significant interaction effects were observed between after the intervention, after 60 min, and 1 week later (Time) and active or sham tDCS (Group). *CI* confidence interval, *Active* active tDCS group, *Sham* sham tDCS group; the lower and upper hinges correspond to the first and third quartiles (25th and 75th percentiles), respectively. *p < 0.05.

The results of the LMMs for STAI-SA showed a significant interaction effect between 1 week after tDCS (Time) and active or sham tDCS (Group) (β = 5.02, SE = 2.13, p = 0.02) (Fig. 2a and Supplementary Table S2 online).

The main effect of Time was significantly different at each assessment time point (immediately after stimulation: β = –5.52, SE = 1.07, p < 0.01; 60 min after stimulation: β = –9.85, SE = 1.07, p < 0.01; and 1 week later: β = –8.67, SE = 1.07, p < 0.01).

The results of the LMMs for STAI-TA showed a significant main effect (Time) after 60 min (β = –1.44, SE = 0.52, p < 0.01) and after 1 week of stimulation (β = –2.72, SE = 0.52, p < 0.01). We did not find the significant interaction between groups and each time point (Fig. 2b and Supplementary Table S3 online), but the interaction effect between groups and 1 week after intervention was marginally significant, a consistent trend with STAI-SA (β = 1.95, SE = 1.03, p = 0.06). As anxiety is known to decrease with increasing age^33,34^, we examined the impact of age on STAI scores at baseline as well as during the course of the intervention. We found no significant correlation between baseline STAI scores and age (STAI-SA, rho = –0.14, p = 0.29; STAI-TA, rho = –0.09, p = 0.51). We also examined whether age had any impact on the outcome of the intervention. We included age groups (20–39 and 40–60 years) used as a stratification factor as a dummy repressor to the LMM (Supplementary Table S4 and S5 online). We found that age was not associated with STAI scores (STAI-SA model: β = 1.44, p = 0.34; STAI-TA mode: β = –1.75, p = 0.52), and that the tDCS effect on STAI scores did not interact with age (STAI-SA model: β = 0.35, p = 0.91; STAI-TA model: β = –0.95, p = 0.86). Age was not associated with STAI scores at 1 week after the intervention (STAI-SA model: β = –1.17, p = 0.51; STAI-TA model: β = 0.40, p = 0.64). Moreover, age was not associated with STAI scores at 1 week after the intervention in the active tDCS group (STAI-SA model: β = –0.07, p = 0.98; STAI-TA model: β = –1.31, p = 0.44). These results suggested that age was not associated with anxiety in the present study cohort.

When analysing PANAS-PA and PANAS-NA scores, one case was missing in the active tDCS group because of an omission in writing (n = 53, real = 26, sham = 27). The results of the LMMs for PANAS-PA did not show any main or interaction effects (Supplementary Table S6 online).

The results of the LMMs for PANAS-NA showed a significant interaction effect between immediately after tDCS (Time) and active or sham tDCS (Group) (β = –3.25, SE = 1.46, p = 0.03) (Supplementary Table S7 online). In addition, the main effect (time) was significant at each assessment time point (immediately after stimulation: β = –2.19, SE = 0.73, p < 0.01; 30 min after stimulation: β = –4.54, SE = 0.73, p < 0.01; 60 min after stimulation: β = –5.41, SE = 0.73, p < 0.01). These results indicated that PANAS-NA scores were higher in the active stimulation group immediately after tDCS-TW-FM.

Next, we compared total scores on the SEIQoL-DW between the active and sham stimulation groups using a two-sample *t*-test. No significant difference was found between post- and pre-intervention (t = 0.79, df = 52, p = 0.43).

We then compared each FFMQ scale between the active and sham stimulation groups using the Wilcoxon signed-rank test. No significant differences were found between groups (Observing: W = 335.5, p = 0.62, Describing: W = 449.5, p = 0.14, Awareness: W = 351.0, p = 0.82, Nonreacting: W = 372.0, p = 0.89, Nonjudging: W = 359.5, p = 0.94).

We also calculated the rACC and left DLPFC current density in the active and sham stimulation groups, respectively (Table 3).

**Table 3:** Results of rACC and lDLPFC alpha current density.

	Overall (*N* = 54)	Active tDCS + TW-FM group (*N* = 26)	Sham tDCS + TW-FM group (*N* = 28)
**Current density in rACC, mean (SD)**			
Pre-intervention	0.79 (0.49)	0.71 (0.41)	0.86 (0.55)
Post-intervention	0.69 (0.48)	0.56 (0.37)	0.81 (0.55)
**Current density in lDLPFC, mean (SD)**			
Pre-intervention	0.76 (0.40)	0.81 (0.43)	0.70 (0.39)
Post-intervention	0.67 (0.29)	0.63 (0.27)	0.70 (0.30)

*rACC* rostral anterior cingulate cortex, *lDLPFC* left dorsolateral prefrontal cortex, *DCS*  transcranial direct current stimulation, *TW-FM* treadmill walking for focused mindfulness.

**Figure 3:**
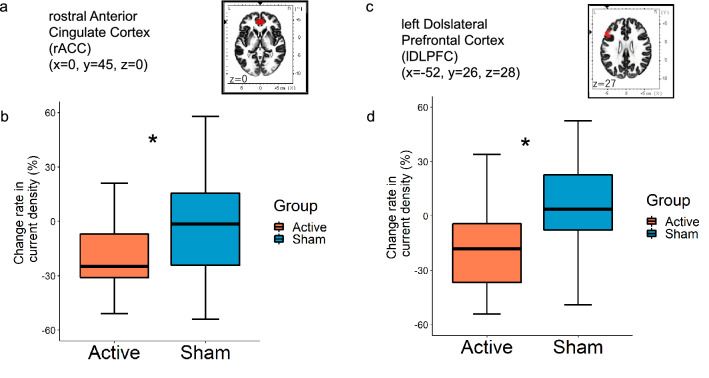
Change rate in current density in the rostral anterior cingulate cortex (rACC) and left dorsolateral prefrontal cortex (lDLPFC). (**a**) Location of regions of interest (rACC). (**b**) A significant reduction in current density was seen in the active compared with the sham stimulation group. The lower and upper hinges correspond to the first and third quartiles (25th and 75th percentiles), respectively. *p < 0.05. (**c**) Location of regions of interest (lDLPFC). (**d**) A significant reduction in current density was seen in the active compared with the sham stimulation group, the lower and upper hinges correspond to the first and third quartiles (25th and 75th percentiles), respectively. *p < 0.05.

To evaluate the effects of the stimulation in the rACC, repeatedly shown its association with an outcome predictor for MDD treatment^22^, we calculated the rate of change through the stimulation in both the active and sham groups. The Mann–Whitney test showed a significantly lower rate in the active compared with the sham group (U = 227·000, p = 0·02) (Fig. 3a,b). Raw current density values in the rACC were then compared between the pre- and post-intervention. The current density was significantly lower at post- than at pre-stimulation in the active stimulation group (Wilcoxon signed-rank test: V = 320, p < 0.001), whereas no significant difference was found between post- and pre-intervention in the sham group (Wilcoxon signed-rank test: V = 226, p = 0.61).

We also examined the left DLPFC current density in the active and sham stimulation groups (Fig. 3c,d) (Table 3) to evaluate changes in activity at the tDCS site. The Mann–Whitney test showed a significantly lower rate in the active compared with the sham group (U = 217, p = 0.006). Raw current density values were significantly lower at post- than at pre-stimulation in the active stimulation group (Wilcoxon signed-rank test: V = 281, p < 0.01), whereas no significant difference was found between post- and pre-intervention in the sham stimulation group (Wilcoxon signed-rank test: V = 156, p = 0.29).

We also examined correlations between the rate of change in STAI scores from before to 1 week after tDCS-TW-FM and in the rACC current density from pre- to post-intervention in each group.

**Figure 4:**
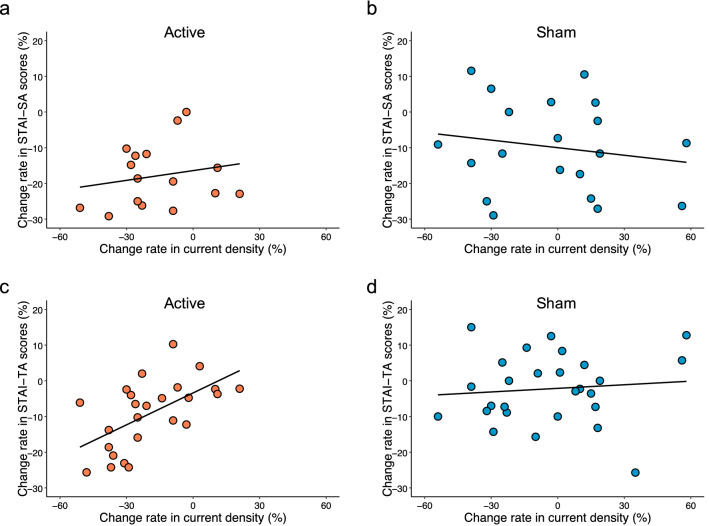
Scatterplot of changes in State-Trait Anxiety Inventory-State Anxiety (STAI-SA) scores (**a**) in the active stimulation group and (**b**) sham stimulation group, and in State Trait Anxiety Inventory-Trait Anxiety (STAI-TA) scores **c** in the active stimulation group and (**d**) sham stimulation group. (**a**) No significant correlation was found (ρ = 0.26, S = 2166, p = 0.20). (**b**) No significant correlation was found (ρ = –0.10, S = 4010, p = 0.62). (**c**) Spearman’s rank correlation coefficient showed a significant correlation between changes in STAI-TA scores and changes in current density in the active stimulation group (ρ = 0.63, S = 1083, p < 0.01). (**d**) No significant correlation was found (ρ = 0.024, S = 3567, p = 0.90).

Spearman’s rank correlation showed no significance between the rate of changes in STAI-SA scores or current density in either the active stimulation on rACC group (ρ = 0.26, S = 2166, p = 0.20) (Fig. 4a) or sham group (ρ = –0.10, S = 4010, p = 0.62) (Fig. 4b).

By contrast, Spearman’s rank correlation coefficient identified a significant correlation between the rates of change in STAI-TA scores and current density in the active stimulation group (ρ = 0.63, S = 1083, p < 0.01) (Fig. 4c); however, no significant correlation was seen in the sham stimulation or rACC group (ρ = 0.024, S = 3567, p = 0.90) (Fig. 4d).

No serious adverse events were reported. Mild adverse events included five cases of skin redness (active group: 3 and sham group: 2), 35 cases of mild pain at the electrode site, (active group: 15 and sham group: 20), and three cases of slight discomfort (active group: 2 and sham group: 3). One participant in the sham stimulation group complained of a headache 2 days after the stimulation that persisted for 2 days before disappearing.

## Discussion

The results of this study revealed an interaction effect between tDCS and time, and that STAI-SA scores were significantly decreased at 1 week after active tDCS. Additionally, we found that the current density of alpha activity in the rACC was significantly decreased in the active tDCS group only, and this reduction was significantly larger in the active than in the sham tDCS group.

Furthermore, in the active stimulation group, the rate of change in STAI-TA scores after 1 week was correlated with the rate of change in rACC alpha activity after tDCS. However, no correlation was observed between STAI-TA scores and rACC alpha activity in the sham stimulation group. These results suggest that immediate neurophysiological changes in the rACC induced by tDCS could reduce anxiety after 1 week.

The rACC is one of the areas of the default mode network, and is considered an important hub in psychiatric disorders associated with anxiety and depression^35,36^. Since the activity of the alpha band is correlated with the default mode network, alpha-band activity in the rACC can be one indicator reflecting the state of the default mode^37^. Stimulation on the DLPFC affects rACC activity^38^. In addition, intensive meditation has been shown to increase DLPFC activity, which suggests that the combination of tDCS and TW-FM would boost DLPFC activity and thereby effectively decrease rACC activity^39^. We also found a correlation between changes in rACC activity immediately after the intervention and changes in STAI-TA scores at 1 week after the intervention in the active but not the sham stimulation group. These results suggest that a combination of TW-FM and tDCS could decrease anxiety by reducing rACC activity, which can help predict the effectiveness of intervention outcomes.

We found a significant reduction in STAI-SA scores after 1 week of the combination of TW-FM and active tDCS. This delayed effect was beyond the scope of our study. On the contrary, PANAS-NA scores immediately after the intervention were increased in the active tDCS group. Although this study had a double-blind design, the participants could feel discomfort as a result of the tDCS. The findings from a previous study support our result regarding a greater perception of discomfort in the active than in the sham stimulation group^40^. It is important to note that despite feeling slightly uncomfortable immediately after the stimulus, a reversal of the response occurred (e.g., a decrease in anxiety) after sufficient time had passed. Another study reported a single tDCS effect later in the day of administration^41^. That study compared the effects of a cognitive training test between online and offline tDCS groups and found no difference on day 1; however, on day 2, the performances were significantly better for the online than for the offline tDCS group. In the present study, we did not measure STAI scores between 1 h and 1 week after tDCS and TW-FM. Therefore, we cannot determine when the reduction in anxiety started. This issue needs to be clarified in a future study.

Regarding STAI scores, we also found a general reduction in scores regardless of intervention type (sham or active). We consider that this was due to general relaxation after the experiments. Indeed, a visual analogue scale (VAS) to assess relaxation showed a significant increasing trend during the course of study, regardless of intervention type (Supplementary Table S1).

Anxiety decreases with increasing age in the general population^33^. However, the prevalence of subgroups of anxiety disorders are quite heterogeneous^42,43^. Our data also showed no significant association between age and STAI scores or intervention effects in our subclinical cohort, but the cohort size was not large enough to perform further stratified analyses. Future research is needed to address how age interacts with intervention outcomes in clinical cohorts.

A broad range of ages may also affect the heterogeneity of physical strength. To minimize these effects, we set the speed of the treadmill machine very slow (1 km/h) to allow the participants to concentrate on their movements. We also found that age was not associated with subjective fatigue after TW-FM (r = –0.02, p = 0.87). These results suggest that age was not a major factor impacting the outcome of our concurrent TW-FM and tDCS intervention.

In the present study, the association among STAI-SA, STAI-TA, and rACC was complex. First, we found that tDCS application during TW-FM can decrease STAI-SA and STAI-TA scores, but STAI-TA only marginally. Second, STAI-SA scores were significantly decreased immediately after the intervention, regardless of intervention type. Third, we found a positive correlation between changes in the rACC current density and STAI-TA scores. These results are indeed puzzling. As defined, the STAI-SA score measures instantaneous changes in anxiety, while the STAI-TA measures the state of anxiety for the last 1 week. Because participants were stressed before the intervention, the STAI-SA score was increased at baseline. This could explain why STAI-SA scores decreased immediately after the intervention, as the participants felt relief from their psychological stress during the intervention. Indeed, we measured relaxation using a VAS; the results revealed that relaxation was highest before and decreased after the intervention (Supplementary Table S1). This may explain why we observed a more robust tDCS effect on the changes in STAI-SA compared with STAI-TA scores. By contrast, we did not observe an immediate reduction in STAI-TA scores immediately after the intervention. Instead, the reduction of rACC alpha activity was significantly correlated with the reduction in STAI-TA scores 1 week after the intervention.

Regarding the stimulation target, in the present study, we applied tDCS to the ventroanterior part of the DLPFC, which is considered more effective for rTMS treatment in MDD^28^. However, it remains unclear which brain region is the most effective site to reduce anxiety. Therefore, similar to the optimization of brain stimulation in the treatment for MDD^44^, future research is needed to optimize stimulation targets in mindfulness as well as anxiety disorders.

In the present study, we applied tDCS on the DLPFC while performing TW-FM; as a result, anxiety was reduced 1 week later. Using EEG source analysis, we found that tDCS reduced alpha activity in the DLPFC and rACC. As repeatedly shown in previous studies predicting the outcomes of MDD treatment with various intervention methods^45,46^, decreased rACC alpha activity is a predictor of better treatment outcomes. We also found that changes in rACC alpha activity were positively correlated with changes in STAI-TA scores at 1 week after the intervention. rACC anatomically connected with the orbitofrontal cortex and amygdala^47^, suggesting that immediate changes in rACC alpha activity can modulate amygdala activity over the long term. As a result, the STAI-TA score measuring anxiety for the last 1 week would be decreased. Although these results fill some gaps, many remain, such as how DLPFC alpha activity affects rACC activity and how rACC alpha activity reduces anxiety over the long term. Future research is needed to clarify these mechanisms.

Several studies have been conducted on the combination of tDCS and mindfulness. One combined tDCS and mindfulness-based training to enhance working memory and another investigated the therapeutic effects of a combination of tDCS and mindfulness-based relapse prevention on alcohol dependence^48–50^.

Other studies have investigated emotion and mood specifically. Badran and colleagues found a significant increase in the “Awareness” item on the FFMQ in a 1-mA compared with a 2-mA stimulation group and a sham group^7^. In the present study, we did not find a significant difference between the sham and active tDCS groups in “Awareness”; however, their result may support the notion that 1 mA is suitable for a combination of mindfulness and tDCS. Another recent study compared MBCT and non-MBCT relaxation in treatment-resistant depression^51^. That study confirmed that the combination of MBCT and tDCS resulted in more sustained clinical improvement in the later period (25 days after the first intervention day, totally after nine sessions). Those results suggest the long-period effect of a combination of mindfulness and tDCS.

In the present study, eligible participants did not have any history or current episode of psychiatric disorders, but at baseline, six of the 54 participants were classified as having high anxiety and 20 as having moderate anxiety according to the STAI-SA, whereas eight were classified as having high anxiety and 14 as having moderate anxiety according to the STAI-TA. Approximately, half of the participants has moderate or high anxiety, while None of them met the criteria for anxiety disorders. Therefore, we consider the substantial participants are implicated in subclinical anxiety.

This study had some limitations. First, other than the day of the intervention, anxiety was assessed only at 1 week after the intervention. Therefore, we could not assess when the reduction of anxiety started and how long it lasted. Future studies need to address this issue over a longer time course of anxiety changes. The second limitation is the intensity of tDCS. Recent studies have used higher-intensity tDCS (2 mA or more), but in this study, we used 1 mA so as to not disturb the mindfulness meditation through discomfort caused by tDCS-induced skin sensations. Third, this study was conducted on a subclinical cohort. Therefore, whether a combination of TW-FM and tDCS is effective for general patients with anxiety disorders remains unclear.

This study revealed that a combination of TW-FM and tDCS reduced alpha-band activity in the rACC immediately after the intervention, and this reduction in anxiety was sustained 1 week later. Despite being a one-shot and short intervention, the reduction in anxiety persisted for at least for 1 week, and EEG could potentially help predict its effects. These findings suggest that a combination of TW-FM and tDCS could improve quality of life for individuals experiencing excessive stress in modern society.

## Supplementary Information

Supplementary Information.

## Data Availability

Individual participant data that underlie the results reported in this article as well as the study protocol are available for the purpose of meta-analysis upon reasonable request. As the disclosure requires additional review and approval by the ethics committee at Kansai Medical University, and additional consent from the study participants, proposals may be submitted to the corresponding author (Dr. Keiichiro Nishida, nishidak@takii.kmu.ac.jp) up to 36 months following publication. Costs to obtain approval of the ethics committee and consent from the participants must be covered by the researcher requesting the individual participant data.
