# Supplementary material for: Mindfulness augmentation for anxiety through concurrent use of transcranial direct current stimulation: a randomized double-blind study
Source: Sci Rep. 2021 Nov 23;11:22734. doi: 10.1038/s41598-021-02177-3 (PMC8610980; doi:10.1038/s41598-021-02177-3)
Supplement: Supplementary file 1 — Supplementary Information. [file 41598_2021_2177_MOESM1_ESM.docx]

**Mindfulness augmentation for anxiety through concurrent use of transcranial direct current stimulation: a randomized double-blind study**

**Supplementary information**

Keiichiro Nishida MD,PhD, Yosuke Morishima MD,PhD, Roberto D. Pascual-Marqui PhD, Shota Minami MD, Tomonari Yamane MEd, Masahito Michikura PhD, Hideki Ishikawa MD,PhD, Toshihiko Kinoshita MD,PhD

**Supplementary Figure S1: CONSORT flow diagram Supplementary**

Consort Flow diagram: Based on the “CONSORT 2010 Flow Diagram” (http://www.consort-statement.org).

Randomized (n=60)

intervention

Allocation

Analyzed (n=29)

♦ Excluded from analysis (poor quality EEG) (n=1)

Analyzed (n=27)

♦ Excluded from analysis (poor quality EEG) (n=1)

Enrollment

Follow-Up

Randomized to Sham stimulation (n=30)

♦ Received allocated intervention (n=30)

Analyzed (n=28)

♦ Failed to submit information at 1 week (n=1)

Randomized to Active stimulation group (n=30)

♦ Received allocated intervention (n=28)

♦ Did not receive allocated intervention (withdrew) (n=2)

Analyzed (n=26)

♦ Failed to submit information at 1 week (n=1)

Assessed for eligibility (n=60)

**Supplementary Table 1. Summary of the psychological tests.**

|  | **Overall (*N*=54)** | **Active tDCS + TW-FM group (*N*=26)** | **Sham tDCS + TW-FM group (*N*=28)** |
| --- | --- | --- | --- |
| STAI-SA, mean (SD) |  |  |  |
| Before the intervention | 43.35 (7.43) | 42.96 (7.73) | 43.71 (7.26) |
| After the intervention | 37.83 (7.22) | 38.85 (8.32) | 36.89 (6.03) |
| 60 min later | 33.50 (5.35) | 33.62 (5.26) | 33.39 (5.53) |
| 1 week later | 34.69 (8.88) | 31.69 (6.13) | 37.46 (10.17) |
|  |  |  |  |
| STAI-TA, mean (SD) |  |  |  |
| Before the intervention | 44.09 (9.64) | 42.88 (7.81) | 45.21 (11.10) |
| After the intervention | 43.19 (9.59) | 41.54 (8.62) | 44.71 (10.34) |
| 60 min later | 42.65 (10.37) | 41.04 (9.15) | 44.14 (11.35) |
| 1 week later | 41.37 (10.33) | 39.15 (8.97) | 43.43 (11.23) |
|  |  |  |  |
| PANAS-PA, mean (SD) |  |  |  |
| Before the intervention | 16.56 (6.40) | 15.54 (5.22) | 17.50 (7.30) |
| After the intervention | 17.26 (6.39) | 17.39 (6.81) | 17.14 (6.10) |
| 30 min later* | 17.09 (7.04) | 16.62 (7.95) | 17.56 (6.17) |
| 60 min later | 17.22 (7.75) | 16.73 (8.45) | 17.68 (7.15) |
|  |  |  |  |
| PANAS-NA, mean (SD) |  |  |  |
| Before the intervention | 16.59 (6.69) | 16.39 (6.178) | 16.786 (7.23) |
| After the intervention | 14.41 (5.28) | 15.89 (5.743) | 13.04 (4.49) |
| 30 min later* | 12.13 (3.97) | 12.35 (4.00) | 11.93 (4.00) |
| 60 min later | 11.19 (4.21) | 11.69 (5.27) | 10.71 (2.94) |
|  |  |  |  |
| SEIQoL-DW, mean (SD) |  |  |  |
| 30 min later | 67.37 (13.37) | 68.87 (13.66) | 65.99 (13.18) |
|  |  |  |  |
| FFMQ, mean (SD) |  |  |  |
| Observing |  |  |  |
| Time of consent | 21.67 (4.56) | 21.65 (5.42) | 21.68 (3.68) |
| 1 week later | 21.94 (5.04) | 21.81 (5.27) | 22.07 (4.91) |
| Describing |  |  |  |
| Time of consent | 23.85 (6.13) | 24.23 (6.08) | 23.50 (6.27) |
| 1 week later | 24.78 (6.38) | 25.65 (5.43) | 23.96 (7.15) |
| Awareness |  |  |  |
| Time of consent | 27.31 (5.56) | 27.00 (5.56) | 27.61 (5.65) |
| 1 week later | 27.37 (5.35) | 27.58 (5.29) | 27.18 (5.50) |
| Nonreacting |  |  |  |
| Time of consent | 21.48 (3.39) | 21.04 (2.51) | 21.89 (4.05) |
| 1 week later | 22.00 (4.16) | 22.15 (3.94) | 21.86 (4.42) |
| Nonjudging |  |  |  |
| Time of consent | 26.48 (5.10) | 26.35 (4.72) | 26.61 (5.51) |
| 1 week later  VAS relaxation, mean (SD)  Time of consent*  Before the intervention*  After the intervention*  60 min later*  Number of days of slow walking with mindfulness*  0  1  2  3  4  5  6  7 | 27.24 (5.40)  5.56 (2.39)  6.30 (1.98)  7.52 (1.74)  7.92 (1.87)  12  12  14  5  3  4  3  1 | 27.04 (4.90)  5.72 (2.18)  6.35 (2.22)  7.41 (1.72)  8.00 (1.57)  6  5  9  3  0  2  1  0 | 27.43 (5.90)  5.40 (2.62)  6.25 (1.76)  7.62 (1.79)  7.84 (2.14)  6  7  5  2  3  2  2  1 |

| tDCS=transcranial direct current stimulation; TW-FM=treadmill walking for focused mindfulness; STAI-SA=State-Trait Anxiety Inventory-State Anxiety; STAI-TA=STAI-Trait Anxiety; PANAS-PA=Positive and Negative Affect Schedule-Positive Affect; PANAS-NA=PANAS-Negative Affect; FFMQ=Five Facet Mindfulness Questionnaire; SEIQoL-DW=Schedule for the Evaluation of the Individual Quality of Life-Direct Weighting; VAS=visual analogue scale. |
| --- |
| * one case was missing in the sham tDCS group. |

**Supplementary table S2. Results of the linear mixed-effects models for State-Trait Anxiety Inventory-State Anxiety (STAI-SA)**

|  | Estimate | Std. Error | df | t value | p-value |  |
| --- | --- | --- | --- | --- | --- | --- |
| **Intercept** | 37·34 | 0·74 | 52 | 50·73 | <0·01 | ** |
| **Time** |  |  |  |  |  |  |
| After Intervention | -5·52 | 1·07 | 156 | -5·18 | <0·01 | ** |
| After 60 min | -9·85 | 1·07 | 156 | -9·24 | <0·01 | ** |
| 1 week later | -8·67 | 1·07 | 156 | -8·13 | <0·01 | ** |
| **Group** | 1·09 | 1·47 | 52 | 0·74 | 0·46 |  |
| **Interaction** |  |  |  |  |  |  |
| After Intervention ×Group | -2·71 | 2·13 | 156 | -1·27 | 0·21 |  |
| After 60 min ×Group | -0·98 | 2·13 | 156 | -0·46 | 0·65 |  |
| 1 week later ×Group | 5·02 | 2·13 | 156 | 2·35 | 0·02 | * |

| ** p<0.01 |
| --- |
| * p<0.05 |

**Supplementary table S3. Results of the linear mixed-effects models for State-Trait Anxiety Inventory-Trait Anxiety (STAI-TA)**

|  | Estimate | Std· Error | df | t value | Pr (>\|t\|) |  |
| --- | --- | --- | --- | --- | --- | --- |
| **Intercept** | 42·82 | 1·32 | 52 | 32·54 | <0·01 | ** |
| **Time** |  |  |  |  |  |  |
| After Intervention | -0·91 | 0·52 | 156 | -1·76 | 0·08 |  |
| After 60 min | -1·44 | 0·52 | 156 | -2·80 | <0·01 | ** |
| 1 week later | -2·72 | 0·52 | 156 | -5·28 | <0·01 | ** |
| **Group** | 3·22 | 2·63 | 52 | 1·22 | 0·23 |  |
| **Interaction** |  |  |  |  |  |  |
| After Intervention ×Group | 0·85 | 1·03 | 156 | 0·82 | 0·41 |  |
| After 60min ×Group | 0·77 | 1·03 | 156 | 0·75 | 0·45 |  |
| 1week later ×Group | 1·95 | 1·03 | 156 | 1·88 | 0·06 |  |

| \| ** p<0.01 \| \| --- \| \| * p<0.05 \| |
| --- | --- | --- |

**Supplementary table S4. Results of the linear mixed-effects models for State-Trait Anxiety Inventory-State Anxiety (STAI-SA) with age**

|  | Estimate | Std. Error | df | t value | p-value |  |
| --- | --- | --- | --- | --- | --- | --- |
| **Intercept** | 37·34 | 0·74 | 50 | 50·22 | <0·01 | ** |
| **Time** |  |  |  |  |  |  |
| After Intervention | -5·52 | 1·07 | 154 | -5·15 | <0·01 | ** |
| After 60min | -9·85 | 1·07 | 154 | -9·19 | <0·01 | ** |
| 1 week later | -8·67 | 1·07 | 154 | -8·09 | <0·01 | ** |
| **Group** | 1·08 | 1·49 | 50 | 0·73 | 0·47 |  |
| **Age** | 1·44 | 1·49 | 50 | 0·97 | 0·34 |  |
| **Interaction** |  |  |  |  |  |  |
| Group × Age | 0·35 | 2·98 | 50 | 0·12 | 0·91 |  |
| 1 week later × Age | -1·17 | 1·76 | 154 | -0·66 | 0·51 |  |
| After Intervention × Group | -2·71 | 2·15 | 154 | -1·26 | 0·21 |  |
| After 60min × Group | -0·98 | 2·15 | 154 | -0·46 | 0·65 |  |
| 1 week later × Group | 5·02 | 2·15 | 154 | 2·34 | 0·02 | * |
| 1 week later × Group × Age | -0·07 | 3·51 | 154 | -0·02 | 0·98 |  |

| ** p<0·01 |
| --- |
| * p<0·05 |

**Supplementary table S5. Results of the linear mixed-effects models for State-Trait Anxiety Inventory-Trait Anxiety (STAI-TA) with age**

|  | Estimate | Std· Error | df | t value | p-value |  |
| --- | --- | --- | --- | --- | --- | --- |
| **Intercept** | 42·82 | 1·34 | 50 | 32·05 | <0·01 | ** |
| **Time** |  |  |  |  |  |  |
| After Intervention | -0·91 | 0·52 | 154 | -1·75 | 0·08 |  |
| After 60min | -1·44 | 0·52 | 154 | -2·79 | 0·01 | ** |
| 1 week later | -2·72 | 0·52 | 154 | -5·25 | <0·01 | ** |
| **Group** | 3·23 | 2·67 | 50 | 1·21 | 0·23 |  |
| **Age** | -1·75 | 2·68 | 50 | -0·65 | 0·52 |  |
| **Interaction** |  |  |  |  |  |  |
| Group × Age | -0·95 | 5·36 | 50 | -0·18 | 0·86 |  |
| 1 week later × Age | 0·40 | 0·85 | 154 | 0·48 | 0·64 |  |
| After Intervention × Group | 0·85 | 1·04 | 154 | 0·82 | 0·42 |  |
| After 60min × Group | 0·77 | 1·04 | 154 | 0·75 | 0·46 |  |
| 1 week later × Group | 1·94 | 1·04 | 154 | 1·88 | 0·06 |  |
| 1 week later × Group × Age | -1·31 | 1·70 | 154 | -0·77 | 0·44 |  |

| ** p<0·01 |
| --- |
| * p<0·05 |

**Supplementary table S6. Results of the linear mixed-effects models for Positive and Negative Affect Schedule Positive Affect**

|  | Estimate | Std. Error | df | t value | Pr (>\|t\|) |  |
| --- | --- | --- | --- | --- | --- | --- |
| **(Intercept)** | 16·99 | 0·86 | 51·94 | 19·83 | <0·01 | ** |
| **Time** |  |  |  |  |  |  |
| After Intervention | 0·70 | 0·67 | 154·94 | 1·06 | 0·29 |  |
| After 30 min | 0·38 | 0·67 | 155·03 | 0·57 | 0·57 |  |
| After 60 min | 0·67 | 0·67 | 154·94 | 1·00 | 0·32 |  |
| **Group** | -0·82 | 1·71 | 51·93 | -0·48 | 0·63 |  |
| **Interaction** |  |  |  |  |  |  |
| After Intervention ×Group | 2·20 | 1·33 | 154·94 | 1·66 | 0·10 |  |
| After 30 min ×Group | 1·34 | 1·34 | 155·03 | 1·00 | 0·32 |  |
| After 60 min ×Group | 1·01 | 1·33 | 154·94 | 0·76 | 0·45 |  |

** p<0·01

**Supplementary table S7. Results of the linear mixed-effects models for Positive and Negative Affect Schedule Negative Affect**

|  | **Estimate** | **Std· Error** | **df** | **t value** | **Pr (>\|t\|)** |  |
| --- | --- | --- | --- | --- | --- | --- |
| (Intercept) | 13·56 | 0·54 | 51·88 | 25·11 | <0·01 | ** |
| Time |  |  |  |  |  |  |
| After Intervention | -2·19 | 0·73 | 154·89 | -2·99 | <0·01 | ** |
| After 30 min | -4·54 | 0·73 | 155·15 | -6·18 | <0·01 | ** |
| After 60 min | -5·41 | 0·73 | 154·89 | -7·41 | <0·01 | ** |
| Group | 1·00 | 1·08 | 51·87 | 0·92 | <0·01 | ** |
| Interaction |  |  |  |  |  |  |
| After Intervention ×Group | 3·25 | 1·46 | 154·89 | 2·23 | 0·03 | * |
| After 30 min ×Group | 0·97 | 1·47 | 155·13 | 0·66 | 0·51 |  |
| After 60 min ×Group | 1·38 | 1·46 | 154·89 | 0·94 | 0·35 |  |

| ** p<0·01 |
| --- |
| * p<0·05 |
